# Left atrial reverse remodeling improves risk stratification in patients with heart failure with recovered ejection fraction

**DOI:** 10.1038/s41598-022-08630-1

**Published:** 2022-03-16

**Authors:** Masayuki Shiba, Takao Kato, Takeshi Morimoto, Hidenori Yaku, Yasutaka Inuzuka, Yodo Tamaki, Neiko Ozasa, Yuta Seko, Erika Yamamoto, Yusuke Yoshikawa, Takeshi Kitai, Yugo Yamashita, Moritake Iguchi, Kazuya Nagao, Yuichi Kawase, Takashi Morinaga, Mamoru Toyofuku, Yutaka Furukawa, Kenji Ando, Kazushige Kadota, Yukihito Sato, Koichiro Kuwahara, Takeshi Kimura

**Affiliations:** 1 Department of Cardiovascular Medicine, Kyoto University Graduate School of Medicine, 54 Shogoin Kawahara-cho, Sakyo-ku, Kyoto, 606-8507 Japan; 2 Department of Clinical Epidemiology, Hyogo College of Medicine, Nishinomiya, Japan; 3 Department of Cardiology, Mitsubishi Kyoto Hospital, Kyoto, Japan; 4 Cardiovascular Medicine, Shiga General Hospital, Moriyama, Japan; 5 Division of Cardiology, Tenri Hospital, Tenri, Japan; 6 Division of Heart Failure, National Cerebral and Cardiovascular Center, Suita, Japan; 7 Department of Cardiology, National Hospital Organization Kyoto Medical Center, Kyoto, Japan; 8 Department of Cardiology, Osaka Red Cross Hospital, Osaka, Japan; 9 Department of Cardiology, Kurashiki Central Hospital, Kurashiki, Japan; 10 Department of Cardiology, Kokura Memorial Hospital, Kokura, Japan; 11 Department of Cardiology, Japanese Red Cross Wakayama Medical Center, Wakayama, Japan; 12 Department of Cardiovascular Medicine, Kobe City Medical Center General Hospital, Kobe, Japan; 13 Department of Cardiology, Hyogo Prefectural Amagasaki General Medical Center, Amagasaki, Japan; 14 Department of Cardiovascular Medicine, Shinshu University Graduate School of Medicine, Nagano, Japan

**Keywords:** Prognostic markers, Outcomes research, Heart failure

## Abstract

We aimed to investigate the relationship between left atrial (LA) reverse remodeling and prognosis of heart failure (HF) with recovered ejection fraction (EF) (HFrecEF). Among 1,246 patients with acute heart failure enrolled in the prospective longitudinal follow-up study, 397 patients with HF with mildly-reduced EF and with reduced EF at discharge were analyzed. Echocardiography was performed during the index hospitalization and at the 6-month follow-up after discharge. They were divided into non-HFrecEF (n = 227) and HFrecEF (n = 170) groups. The primary outcome measure was a composite of all-cause death or hospitalization for HF. The cumulative 180-day incidence of the primary outcome measure after follow-up echocardiography was significantly lower in the HFrecEF group than in the non-HFrecEF group (8.9% versus 23.4%, log-rank P = 0.0002). LA reverse remodeling was associated with a lower cumulative 6-month incidence of the primary outcome measure in the HFrecEF group (4.7% versus 18.0%; HR: 0.27, 95%CI: 0.09–0.79, P = 0.01), but not in the non-HFrecEF group (24.4% versus 22.6%; HR: 1.13, 95%CI: 0.65–1.96, P = 0.28) with a significant LA reverse remodeling-by-HFrecEF interaction (P for interaction = 0.02). Combination of left ventricular and atrial reverse remodeling may help in improving HF risk stratification.

## Introduction

Categorization of heart failure (HF) according to change in left ventricular ejection fraction (LVEF) is one of the current topics in HF^1,2^. HF with recovered ejection fraction (HFrecEF) has not been definitively established with several different HFrecEF definitions such as HF in a patient with reduced LVEF in the past but with a ≥ 50% improved LVEF and HF in a patient with LVEF < 40% at baseline but with ≥ 10% absolute improvement in LVEF^1–3^. Previous studies showed characteristics, outcomes and factors associated with LVEF improvement in patients with HF with mildly-reduced ejection fraction (HFmrEF) and HF with reduced ejection fraction (HFrEF)^4–6^. Patients with HFrecEF have a substantially better prognosis than those without HFrecEF^7^. Moreover, HFrecEF patients had improved clinical outcomes compared with patients with HF with preserved ejection fraction (HFpEF)^8^. HFrecEF is one of the important phenotypes of HF. However, data on the factors affecting the prognosis of patients with HFrecEF are scarce.

Enlargement of left atrium (LA) is associated with various adverse cardiovascular events and has been established as a prognostic marker for HF^9,10^. Evidence regarding the association between sequential change in LA size and clinical outcomes in HF patients is limited. However, favorable outcomes were observed in patients with LA reverse remodeling^11^. Since LA is structurally and functionally correlated with left ventricular (LV) function^12,13^, we aimed to investigate the relationship between LA reverse remodeling and prognosis of patients with HFrecEF.

## Methods

### Patient population

We enrolled 1,246 patients with acute HF (AHF) who were scheduled to visit at 6 ± 1 month into the prospective longitudinal follow-up study parallel with the main Kyoto Congestive Heart Failure (KCHF) registry. The rationale, design and enrolment of the KCHF registry have been previously published in detail^14,15^. Detailed enrollment of 1,246 patients is included in the Supplementary Method. Of 748 patients who underwent follow-up echocardiography at 6 months after discharge, the current study population consisted of 397 patients with HFmrEF and HFrEF who had serial echocardiographic data on change in LVEF or left atrial diameter (LAD) from index hospitalization to 6 months follow-up (Supplementary Fig. 1).

### Ethics

The investigation conformed to the principles outlined in the Declaration of Helsinki. The study protocol was approved by the ethical committees of the Kyoto University Hospital (local identifier: E2311) and in each participating hospital. Written informed consent was obtained from the patients enrolled in the longitudinal prospective cohort study.

### Outcomes

Clinical follow-up was conducted at 1 year ± 1 month after enrolment; thus, the date of the 6-month follow-up echocardiography was considered as time zero for evaluating the clinical events censored at 210 days after the follow-up echocardiography in this study (Supplementary Fig. 2). The participating physicians or research assistants at each participating hospital collected data on clinical events after the index hospitalization from medical records or by contacting patients, their relatives, or their referring physicians. The primary outcome measure in this study was defined as a composite endpoint of all-cause death or HF hospitalization^15^. The definitions of causes of death are described in Supplementary Methods.

### Definitions

We recommended echocardiographic measurements including LVEF and LAD by taking the average of three beats for patients with normal sinus rhythm and a minimum of five beats in patients with atrial fibrillation^16,17^; nonetheless, we designed the use of representative beats acceptable according to the two-chamber quantification guidelines^16,17^. In the present study, HFrecEF was defined as absolute LVEF improvement ≥ 10% between index hospitalization and 6-month follow-up echocardiography in patients with HFmrEF and HFrEF^3^. LAD reduction was calculated as (LAD during index hospitalization-LAD at follow-up echocardiography)/ LAD during index hospitalization × 100 (%). LA reverse remodeling was defined as LAD reduction ≥ 5% at follow-up echocardiography^18^. Patients were classified according to LVEF at index hospitalization (HFpEF: LVEF ≥ 50%, HFmrEF: LVEF 40%–49% and HFrEF: LVEF < 40%). The detailed definitions of baseline patient characteristics have been previously described^14,15^. Anemia was defined using the World Health Organization criteria (hemoglobin < 120 g/L in women and < 130 g/L in men). End-stage renal disease was defined as an estimated glomerular filtration rate < 30 mL/min/1.73 m^2^ based on the chronic kidney disease grades. Atrial arrythmias included atrial fibrillation or atrial flutter. A medical history of prior myocardial infarction (MI) at the 6-month visit, which was one of 11 adjusting variables, was counted based on past history at index hospitalization except for acute coronary syndrome (ACS) during index hospitalization because we designed ACS as one of the exclusion criteria for the follow-up study.

### Statistical analysis

Continuous variables were expressed as mean and standard deviations or medians with interquartile ranges and categorical variables are expressed as counts and percentages. For comparisons between groups, categorical variables were compared using χ^2^ test, and continuous variables were compared using unpaired t-test or Wilcoxon rank-sum test. A paired t-test was used for continuous variables and sign test was used for binary variables to compare those at the index hospitalization and those at the 6-month visit. Cumulative incidences were calculated by means of the Kaplan–Meier analysis and the among-groups differences were tested by means of the log-rank test. We used the Cox proportional hazards regression model to estimate the association between HFrecEF and the primary outcome measure after adjusting for 11 clinically relevant risk variables: age ≥ 80 years, sex, atrial arrythmias, MI, anemia, eGFR < 30 ml/min/1.73m^2^, moderate or severe mitral regurgitation (MR), LAD reduction ≥ 5% and medications at follow-up (angiotensin converting-enzyme inhibitor [ACE-I] or angiotensin-receptor blocker [ARB], β-blocker, and mineralocorticoid receptor antagonist [MRA]). The results were expressed as the hazard ratios (HRs) and their 95% confidence intervals (CIs). We evaluated the effect of an LA reverse remodeling-by-HFrecEF interaction by means of the univariate Cox proportional hazards regression model. As a sensitivity analysis, we included a three-group classification of change in LVEF (group 1: < 0%, N = 90, group 2: ≥ 0% and < 10%, N = 137, and group 3: ≥ 10%, N = 170) into the adjusted model with group 1 as the reference and estimated the adjusted risk of group 2 versus group 1 and that of group 3 versus group 1. We used a scatter plot across change in LAD and change in LVEF, using the Pearson correlation coefficient (Pearson’s r) and the simple linear regression model. An absolute value of r > 0.3 or > 0.7 was considered as a moderate or strong relationship, respectively. The multiple linear regression analysis was performed to assess the factors independently associated with increase in LAD. Candidate variables included change in LVEF as well as 12 other variables such as heart rate, LA enlargement associated factors including age, sex, body mass index, atrial arrythmias, hypertension and congestion-associated factors including moderate/severe MR, change in TRPG and use of diuretics and medications, which may directly or indirectly contribute to LA reverse remodeling (ACE-I or ARB, MRA and β-blocker)^19,20^. We performed additional echocardiographic comparisons between the HFrEF and HFmrEF groups. We compared heart rate, LVEF and LAD between atrial arrythmias and non-atrial arrythmias. A detailed method of additional analysis according to heart failure with improved ejection fraction (HFimpEF) was described in the Supplementary Method^21^.

Statistical analyses were performed using JMP Pro software, version 16.1.0 (https://www.jmp.com/en_us/software/predictive-analytics-software.html) (SAS Corp., Cary, NC, USA). A two-tailed P value < 0.05 was considered statistically significant in all analyses.

## Results

### Clinical characteristics, laboratory test results, and medications at 6-month visit in patients with HFrecEF and non-HFrecEF

During follow-up echocardiography at 6 months after discharge, LVEF improvement ≥ 10% was present in 170 patients (HFrecEF) and absent in 227 patients (non-HFrecEF). Compared with patients in the non-HFrecEF group, those in the HFrecEF group were younger and had a lower prevalence of a history of hypertension, diabetes mellitus, dyslipidemia and MI (Table 1). Patients in the HFrecEF group had lower BNP levels, higher eGFR and higher serum albumin levels, and had a higher prevalence of β-blocker use and a lower prevalence of diuretic use at 6 months visit (Table 1).

**Table 1:** Patient characteristics at 6-month echocardiographic follow-up.

Variable	Non-HFrecEF (N = 227)	HfrecEF (N = 170)	P value	No. of patients analyzed
**Clinical characteristics**				
Age (years)	74.6 ± 12.3	68.9 ± 13.6	< 0.0001	397
Age ≥ 80 years^a^	94 (41%)	39 (23%)	< 0.0001	397
Women^a^	74 (47%)	63 (37%)	0.36	397
BMI (kg/m^2^)	22.8 ± 5.2	22.5 ± 4.3	0.63	299
BMI ≤ 22 kg/m^2^	85 (51%)	67 (50%)	0.89	299
**Medical history**				
Atrial fibrillation or flutter^a^	114 (50%)	80 (47%)	0.53	397
Hypertension	167 (74%)	105 (62%)	0.01	397
Diabetes	103 (45%)	46 (27%)	0.0002	397
Dyslipidemia	112 (49%)	51 (30%)	< 0.0001	397
Previous myocardial infarction^a^	85 (37%)	24 (14%)	< 0.0001	397
Previous ischemic stroke or ICH	33 (15%)	15 (8.8%)	0.08	397
Chronic lung disease	32 (14%)	14 (8.2%)	0.07	397
**Tests at 6-month visit**				
BNP (pg/mL)	282.0 (141.1–587.9)	83.5 (27.2–205.2)	< 0.0001	302
eGFR (mL/min/1.73m^2^)	45.3 ± 19.9	52.2 ± 21.5	0.002	374
eGFR < 30 mL/min/1.73m^2^ ^a^	52 (24%)	23 (15%)	0.03	374
Albumin (g/dL)	3.8 ± 0.5	4.1 ± 0.5	< 0.0001	355
Albumin < 3 g/dL	7 (3.4%)	2 (1.3%)	0.19	355
Hemoglobin (g/dL)	12.3 ± 2.0	12.5 ± 2.5	0.40	370
Anemia^a^	126 (58%)	75 (49%)	0.09	370
**Medications at 6-month visit**				
ACE-I or ARB^a^	123 (65%)	97 (69%)	0.42	329
β-blocker^a^	153 (81%)	127 (91%)	0.007	328
MRA^a^	89 (47%)	75 (54%)	0.26	328
Diuretics	170 (90%)	108 (77%)	0.001	330

Diuretics included loop diuretic, thiazide and tolvaptan.

^a^Risk-adjusting variables selected for the Cox proportional hazards regression model.

HFrecEF, heart failure with recovered ejection fraction; BMI, body mass index; ICH, intracranial hemorrhage; BNP, brain natriuretic peptide; eGFR, estimated glomerular filtration rate; ACE-I, angiotensin-converting enzyme inhibitor; ARB, angiotensin-receptor blocker; MRA, mineralocorticoid receptor antagonist.

### Echocardiographic findings during index hospitalization and at 6-month visit

**Figure 1:**
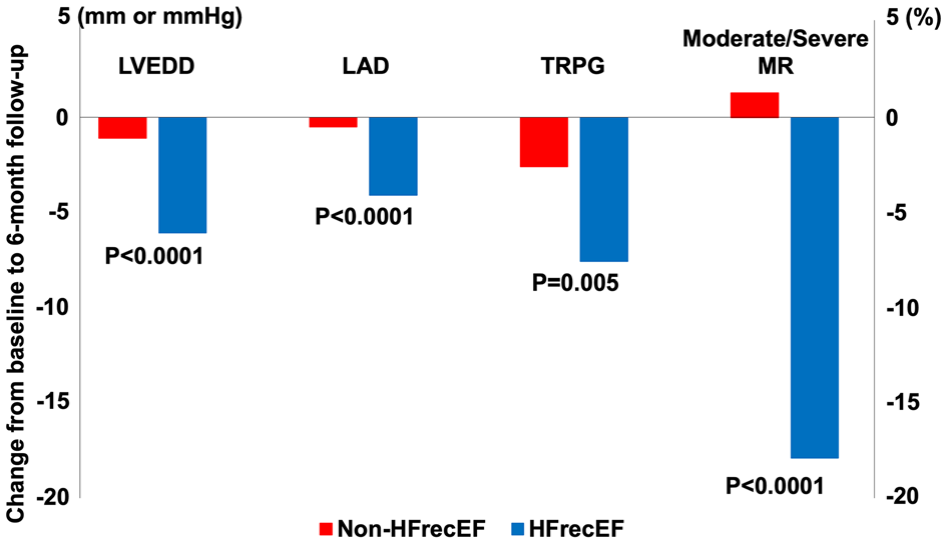
Changes in echocardiographic parameters from index hospitalization to 6-month follow-up echocardiography: HFrecEF versus non-HFrecEF. Figure made utilizing Microsoft Excel 2019, https://products.office.com/. Changes in each echocardiographic parameter are represented as mean values. LVEDD, left ventricular end-diastolic dimension; LAD, left atrial diameter; TRPG, tricuspid regurgitation pressure gradient; MR, mitral regurgitation; HFrecEF, heart failure with recovered ejection fraction.

On echocardiography during index hospitalization, the HFrecEF group had smaller left ventricular end-diastolic dimension (LVEDD) and LAD, lower left ventricular mass index (LVMI) and lower LVEF than the non-HFrecEF group (Supplementary Table 1). At the 6-month follow-up echocardiography, the HFrecEF group had better values for all echocardiographic parameters (Supplementary Table 1). From the index hospitalization to the 6-month follow-up echocardiography, the HFrecEF group compared with the non-HFrecEF group had greater improvement in all the echocardiographic parameters except for moderate/severe tricuspid regurgitation (Supplementary Table 1 and representative values in Fig. 1). LAD reduction ≥ 5% during follow-up was found in 108 patients (64%) in the HFrecEF group, and in 88 patients (39%) in the non-HFrecEF group. Among those patients with LAD reduction ≥ 5% during follow-up, the magnitude of reduction in LAD was greater in the HFrecEF group than in the non-HFrecEF group (− 4.1 ± 6.5 mm versus − 0.5 ± 7.1 mm, P < 0.0001).

### Additional echocardiographic comparison between HFrEF and HFmrEF

This study population was divided into 270 HFrEF patients and 127 HFmrEF patients. During the 6-month follow-up, the HFrEF group had a greater reduction in LVEDD, increase in LVEF, reduction in LAD, reduction in TRPG and reduction in IVC. However, there was no significant difference in prevalence of LVEF improvement ≥ 10% between HFrEF and HFmrEF (Supplementary Table 2).

### Additional comparison of heart rate between atrial arrythmias and non-atrial arrythmias

In the both groups, heart rate at 6 months visit was faster than that at baseline. There was no significant difference in heart rate between the both groups. Change in LVEF was not significantly different; however, change in LAD was significantly different between the both groups (Supplementary Table 3).

### Factors associated with left atrial reverse remodeling from index hospitalization to 6-month follow-up echocardiography

There was a significant correlation between change in LVEF and change in LAD with a weak relationship (β = −0.14, P < 0.0001, R^2^ = 0.066 and Pearson’s r = −0.26) (Supplementary Fig. 3). In the multiple linear regression analysis, atrial arrhythmias and increase in TRPG were positively associated with increase in LAD, while increase in LVEF was negatively correlated with increase in LAD (Table 2).

**Table 2:** Multiple linear regression analysis for increase in LAD.

Variable	Increase in LAD	
	β	P value
Age (years)	0.03	0.46
Women	0.04	0.94
BMI (kg/m^2^)	−0.05	0.61
Heart rate (bpm)	−0.002	0.96
Atrial fibrillation or flutter	1.20	0.006
Hypertension	0.72	0.11
ACE-I or ARB use	0.28	0.56
MRA use	−0.19	0.68
β-blocker use	−0.17	0.79
Diuretics use	0.76	0.27
Increase in LVEF (%)	−0.11	0.002
Moderate/Severe MR	−0.73	0.08
Increase in TRPG (mmHg)	0.06	0.03

Diuretics included loop diuretic, thiazide and tolvaptan.

LAD, left atrial diameter; BMI, body mass index; ACE-I, angiotensin-converting enzyme inhibitor; ARB, angiotensin-receptor blocker; MRA, mineralocorticoid receptor antagonist; LVEF, left ventricular ejection fraction; MR, mitral regurgitation; TRPG, tricuspid regurgitation pressure gradient.

### Clinical outcomes: HFrecEF versus non-HFrecEF

**Figure 2:**
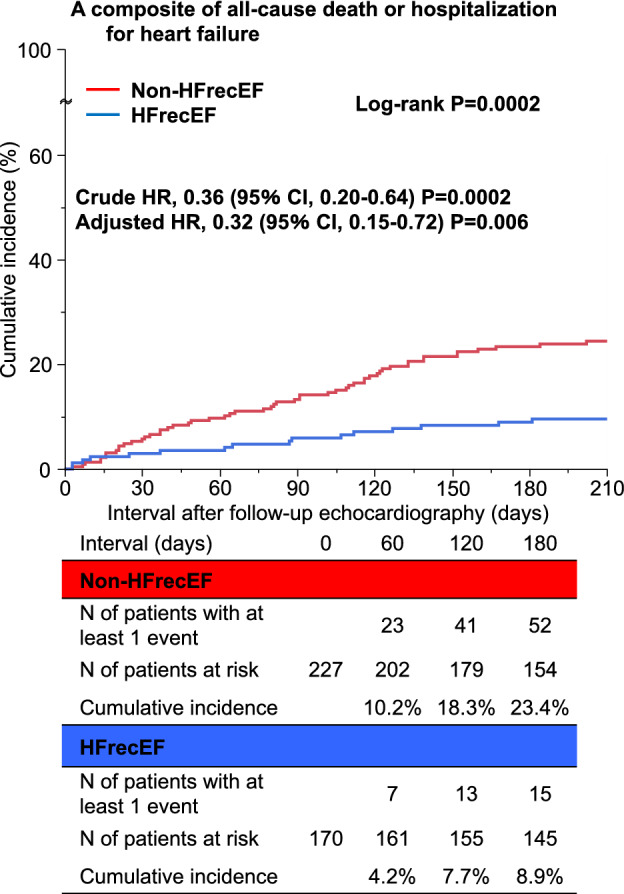
Kaplan Meier curves for a composite of all-cause death or hospitalization for heart failure: HFrecEF versus non-HFrecEF. Figure made utilizing JMP Pro 16.1.0, https://www.jmp.com/en_us/software/predictive-analytics-software.html. HFrecEF, heart failure with recovered ejection fraction; HR, hazard ratio; CI, confidence interval.

The clinical follow-up rate at 180-day after the follow-up echocardiography was 96.5%. The total number of the primary outcome measures was 70 (HF: N = 45, cardiovascular death: N = 19, non-cardiovascular death: N = 6) (Supplementary Fig. 4). The cumulative 180-day incidence of the primary outcome measure of all-cause death or HF hospitalization was significantly lower in the HFrecEF group than in the non-HFrecEF group (8.9% versus 23.4%, log-rank P = 0.0002) (Fig. 2). In multivariable Cox proportional hazard analysis, the lower risk of the HFrecEF group relative to the non-HFrecEF group was significant for the primary outcome measure (HR: 0.32, 95%CI: 0.15–0.72, P = 0.006) (Fig. 2). We performed an additional analysis according to HFimpEF and the results were consistent with those of the main analysis (Supplementary Result and Supplementary Figs. 5 and 6).

### Effect of left atrial reverse remodeling on clinical outcomes based on left ventricular reverse remodeling

**Figure 3:**
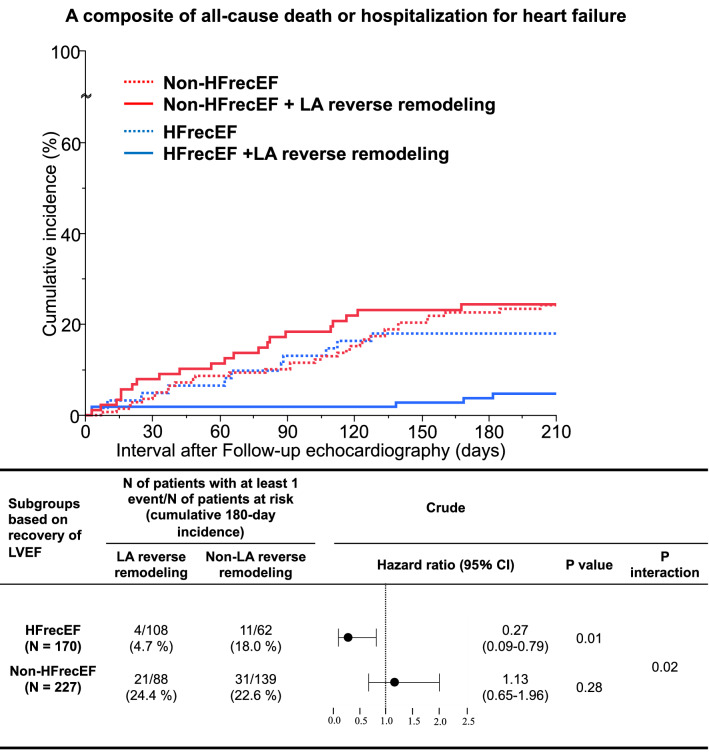
Analysis for a composite of all-cause death or hospitalization for heart failure by the combination of left ventricular and atrial reverse remodeling. Figure made utilizing JMP Pro 16.1.0, https://www.jmp.com/en_us/software/predictive-analytics-software.html. HFrecEF, heart failure with recovered ejection fraction; LVEF, left ventricular ejection fraction; LA, left atrium; CI, confidence interval.

LA reverse remodeling was associated with a lower cumulative 6-month incidence of the primary outcome measure in the HFrecEF group (4.7% versus 18.0%; HR: 0.27, 95%CI: 0.09–0.79, P = 0.01), but not in the non-HFrecEF group (24.4% versus 22.6%; HR: 1.13, 95%CI: 0.65–1.96, P = 0.28) (Fig. 3). There was a significant interaction between LVEF improvement and the effect of LA reverse remodeling relative to no LA reverse remodeling for the primary outcome measure (P for interaction = 0.02) (Fig. 3).

### Sensitivity analyses

The results of the sensitivity analyses were fully consistent with the main results. In the sensitivity analysis based on a three-group classification of change in LVEF (group 1: < 0%, N = 90, group 2: ≥ 0% and < 10%, N = 137, and group 3: ≥ 10%, N = 170), the cumulative 180-day incidence of the primary outcome measure was much lower in group 3 than in the other two groups (group 1: 26.5%, group 2: 21.4%, and group 3: 8.9%, log-rank P = 0.0006) (Supplementary Fig. 7). After adjustment for confounders, the lower risk of the group 3 relative to the group 1 remained significant for the primary outcome measure (HR 0.33, 95% CI 0.14–0.79; P = 0.01) (Supplementary Fig. 8). When we included a three-group classification of change in LVEF into the Cox hazard regression model, there was a significant interaction between change in LVEF and the effect of LA reverse remodeling on the primary outcome measure (P for interaction = 0.047) (Supplementary Fig. 9).

## Discussion

The main findings of the present study are as follows; (1) improvement of LVEF at 6 months after hospitalization for AHF was associated with a lower risk for subsequent death or HF hospitalization; (2) LA reverse remodeling at 6 months after hospitalization for AHF was associated with a lower risk for subsequent death or HF hospitalization in patients with HFrecEF, but not in patients with non-HFrecEF with a significant LA reverse remodeling-by-HFrecEF interaction; and (3) there was a significant correlation between improvement in LVEF and LA reverse remodeling from the index hospitalization to the 6-month follow-up echocardiography.

Many previous studies showed better clinical outcomes of HFrecEF and factors associated with improved LVEF^3–8^. To the best of our knowledge, no previous study focused on the relationship between LA reverse remodeling and LV reverse remodeling in patients with HFmrEF or HFrEF at baseline. Patients with HFrecEF were younger and had a smaller LVEDD, lower prevalence of MI and higher prevalence of β-blocker use, which also improved diastolic function, at the 6-month follow-up echocardiography than those with non-HFrecEF^22^. These findings were consistent with previous studies as factors associated with improved LVEF^3,4,6^. When LV reverse remodeling and LA reverse remodeling were analyzed in combination, LA reverse remodeling was associated with a lower risk for subsequent death or HF hospitalization in patients with HFrecEF, but not in patients with non-HFrecEF with a significant LA reverse remodeling-by-HFrecEF interaction. The impact of LA reverse remodeling may differ according to the concomitant improvement in LVEF.

**Figure 4:**
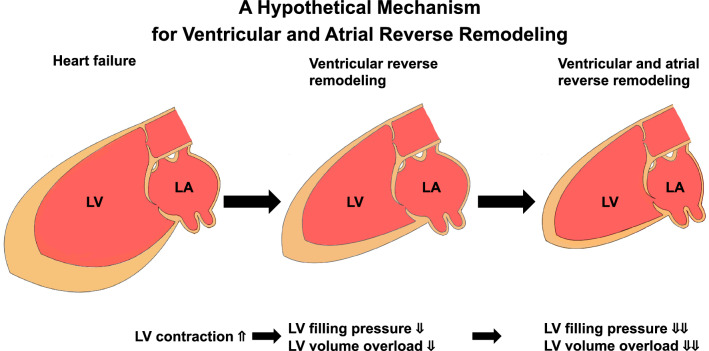
A hypothetical mechanism for ventricular and atrial reverse remodeling. LV, left ventricle; LA, left atrium.

LA conducts as an elastic reservoir for pulmonary venous inflow during LV systole, a passive conduit for pulmonary venous flow during early ventricular diastole and a booster pump to aid LV filling during late ventricular diastole^23^. Conversely, LV filling pressure is backward-transmitted to LA pressure and connected with LA size^12,13^. LA is structurally and functionally correlated with LV function^12,13^. Thus, change in LV filling pressure may be associated with LA size. Compared with the non-HFrecEF group, the HFrecEF group had a greater decrease in LVMI indicating less pressure loading and a greater decrease in TRPG as well as a lower prevalence of moderate/severe MR indicating less volume loading^24^. Altogether, with improved LVEF, a decrease in LV filling pressure and volume loading may be attributed to LA reverse remodeling. Moreover, LV reverse remodeling was closely linked to LA reverse remodeling. Given the significant LA reverse remodeling-by-HFrecEF interaction on clinical outcomes, the presence of LA reverse remodeling might indicate stepwise progress in LV recovery (schematic representation in Fig. 4).

LA reverse remodeling might be a clinically important phenotype of HFrecEF with advanced LV recovery, although our data should be regarded as hypothesis-generating. We might be able to improve HF risk stratification by combining LV and LA reverse remodeling.

## Limitations

This study has several limitations. First, LA reverse remodeling was evaluated by LAD reduction which is not a reliable marker to assess LA size^25^, although LA volume quantification to assess LA size was recommended by guidelines^16,17,26^. In addition, we did not collect data regarding tissue Doppler such as the mitral annulus velocity (E’). Second, heart rate and atrial arrythmias may influence the precision of echocardiographic measurements. Approximately half of this study population had atrial arrythmias. We recommended echocardiographic measurements including LVEF and LAD by taking the average of three beats for patients with normal sinus rhythm and a minimum of five beats in patients with atrial fibrillation according to the two-chamber quantification guidelines^16,17^. Third, there was very significant selection bias. The present study population comprised only 397 patients of the 4,056 patients enrolled in the KCHF registry or of the 1,246 patients scheduled for a 6-month follow-up. In addition, regional differences in HF patients may limit the generalizability of this study. The total 748 patients with follow-up echocardiography consisted of 270 HFrEF patients (40.1%), 127 HFmrEF patients (18.9%), 276 HFpEF patients (41.0%) and 75 patients without data for change in LAD or LVEF. The HFpEF proportion in this study (41.0%) was relatively higher than that of ESC-HF-LT registry, of the Olmsted County cohort study in the USA and the PREVEND study in the Netherlands (25.7%, 28.0% and 34.0%, respectively)^27–29^. However, the HFpEF proportion in this study was not especially high by comparison with other Japanese heart failure cohort studies (CHART-1: 50.6% and CHART-2: 68.7%)^30^. Fourth, although HFmrEF and HFrEF patients were analyzed, the compliance rate of guideline-directed medical therapy for HF, especially MRA was low. Finally, the follow-up period was relatively short and relatively few clinical events occurred in the present study, which made full adjustment difficult. Residual and unmeasured confounding factors may be related to outcomes. Further studies are needed to combine the more precise echocardiographic parameters (i.e. LA volume index) and HFrecEF in one model.

## Conclusion

LA reverse remodeling at 6 months after hospitalization for AHF was associated with a lower risk for subsequent death or HF hospitalization in patients with HFrecEF, but not in patients with non-HFrecEF with a significant LA reverse remodeling-by-HFrecEF interaction, suggesting that the combination of LV and LA reverse remodeling may help us to improve HF risk stratification.

## Supplementary Information

Supplementary Information.

## Data Availability

All data relevant to the study are included in the article or uploaded as supplementary information.
