# Supplementary material for: Left atrial reverse remodeling improves risk stratification in patients with heart failure with recovered ejection fraction
Source: Sci Rep. 2022 Mar 16;12:4473. doi: 10.1038/s41598-022-08630-1 (PMC8927594; doi:10.1038/s41598-022-08630-1)
Supplement: Supplementary file 1 — Supplementary Information. [file 41598_2022_8630_MOESM1_ESM.docx]

**Supplementary material for**

Shiba. et al. Left atrial reverse remodeling improves risk stratification in patients with heart failure with recovered ejection fraction

**Contain methods, results, references, 3 tables, and 9 figures**

**Supplementary Method**

**Enrolled population into the prospective longitudinal follow-up study**

In the Kyoto Congestive Heart Failure registry, we enrolled consecutive 4056 hospitalized patients who were diagnosed as acute heart failure (HF) by the modified Framingham criteria and were treated with HF- specific management involving intravenous drugs within 24 hours after hospital admittance between 1 October 2014 and 31 March 2016 (1, 2). We excluded 271 patients who died during index hospitalization and 2,539 patients corresponding to exclusion criteria from consecutive 4,056 patients. We enrolled 1,246 patients into the prospective longitudinal follow-up study. Exclusion criteria for the prospective longitudinal follow-up study included: no consent given for the longitudinal study (N=238), age <20 years (N=1), fever or infectious diseases at admission (N=297), acute coronary syndrome at admission (N=157), end-stage renal failure (N=218), severe comorbidity which limits the life expectancy within one year assessed by the attending physicians at each participating center, such as end-stage cancer, severe cognitive dysfunction, and end-stage liver dysfunction (N=112), or ineligible for the protocol mandated follow-up by attending physicians’ judgment (N=1,516).

**Definitions of cause of death**

The causes of death were classified according to the VARC (Valve Academic Research Consortium) definitions (3), and were adjudicated by a clinical event committee (1). Death was regarded as cardiovascular in origin unless obvious non-cardiovascular causes could be identified. Cardiovascular death included death related to heart failure, acute myocardial infarction, fatal ventricular arrhythmia, sudden cardiac death, other cardiac death, stroke, intracranial hemorrhage, and other vascular death. Sudden cardiac death was defined as unexplained death of a previously stable patient, including fatal ventricular arrhythmia, and cardiac arrest. Non-cardiovascular death included malignancy, infections, renal failure, liver failure, respiratory failure, bleeding, and other causes. HF hospitalization was due to worsening heart failure, requiring intravenous drug therapy (2).

**Additional analysis according to heart failure with improved ejection fraction**

HF with improved ejection fraction (HFimpEF) was defined as HF with a baseline left ventricular ejection fraction (LVEF) of ≤40%, a ≥10-point increase from baseline LVEF, and a second measurement of LVEF of >40% (4). We analyzed 270 HF with reduce ejection fraction patients and divided them into HFimpEF and non-HFimpEF. Furthermore, we classified them into 4 groups based on HFimpEF/non-HFimpEF and with/without left atrial diameter reduction ≥5%. Cumulative incidences were calculated by means of the Kaplan–Meier analysis and the among-groups differences are tested by means of the log-rank test. We used the Cox proportional hazards regression model to estimate the association between HFimpEF and the primary outcome measure after adjusting for 11 clinically relevant risk variables. The results were expressed as the hazard ratios and their 95% confidence intervals.

**Supplementary Result**

**Additional analyses by HFimpEF**

The results of additional analyses were consistent with the main results. The cumulative 180-day incidence of the primary outcome measure was significantly lower in the HFimpEF group than in the non-HFimpEF group (5.0 % versus 23.6 %, log-rank P=0.0001) (Supplementary Fig. 5). In multivariable Cox proportional hazard analysis, the lower risk of the HFimpEF group relative to the non-HFimpEF group was significant for the primary outcome measure (HR: 0.27, 95%CI: 0.09-0.78, P=0.02) (Supplementary Fig. 5). The cumulative 180-day incidence of the primary outcome measure was much lower in the HFimpEF with LA reverse remodeling than in the other 3 groups (HFimpEF with LA reverse remodeling: 1.6 %, HFimpEF without LA reverse remodeling: 13.5%, Non-HFimpEF with LA reverse remodeling: 18.7%, non-HFimpEF without LA reverse remodeling: 28.0%, log-rank P=0.0002) (Supplementary Fig. 6).

**References for supplementary materials**

1. Yaku H. et al. Demographics, Management, and In-Hospital Outcome of Hospitalized Acute Heart Failure Syndrome Patients in Contemporary Real Clinical Practice in Japan- Observations From the Prospective, Multicenter Kyoto Congestive Heart Failure (KCHF) Registry. *Circ J*. 2018;82(11):2811-9.

2. Yamamoto E. et al. Kyoto Congestive Heart Failure (KCHF) study: rationale and design. *ESC Heart Fail*. 2017;4:216-23.

3. Kappetein AP. et al. Updated standardized endpoint definitions for transcatheter aortic valve implantation: the Valve Academic Research Consortium-2 consensus document. *J Am Coll Cardiol*. 2012;60:1438-54.

4. Bozkurt B. et al. Universal Definition and Classification of Heart Failure. *J Card Fail*. 2021;27(4):387-413.

**Supplementary Table**

**Supplementary Table 1. Changes in echocardiographic parameters from baseline to 6-month follow-up**

|  | **Non-HFrecEF (N=227)** | | | | **HFrecEF (N=170)** | | | | **Between-groups comparison** | | |
| --- | --- | --- | --- | --- | --- | --- | --- | --- | --- | --- | --- |
| Variable | Baseline | Follow-up | Delta ^#^ | P value  (paired) | Baseline | Follow-up | Delta ^#^ | P value  (paired) | P value  (baseline) | P value  (follow-up) | P value  (delta) |
| LVEDD (mm) | 58.4 ± 9.3 | 57.3 ± 9.7 | -1.1 ± 4.5 | 0.0002 | 55.2 ± 7.7 | 49.1 ± 7.0 | -6.1 ± 6.6 | <0.0001 | 0.0002 | <0.0001 | <0.0001 |
| LVESD (mm) | 48.7 ± 10.5 | 47.4 ± 11.0 | -1.3 ± 5.5 | 0.0003 | 46.0 ± 9.1 | 34.9 ± 7.7 | -11.1 ± 7.6 | <0.0001 | 0.007 | <0.0001 | <0.0001 |
| LVMI (g/m^2^) | 143.5 ± 36.1 | 136.9 ± 38.6 | -7.9 ± 27.4 | 0.0003 | 131.2 ± 34.2 | 103.6 ± 29.0 | -28.7 ± 28.8 | <0.0001 | 0.0007 | <0.0001 | <0.0001 |
| LVEF (%) | 34.3 ± 9.5 | 34.7 ± 10.3 | 0.4 ± 5.9 | 0.27 | 32.3 ± 9.9 | 53.9 ± 11.4 | 21.6 ± 9.2 | <0.0001 | 0.048 | <0.0001 | <0.0001 |
| HFrEF | 65 %  (147/227) | n/a | n/a | n/a | 72 %  (123/170) | n/a | n/a | n/a | 0.11 | n/a | n/a |
| HFmrEF | 35 %  (80/227) | n/a | n/a | n/a | 28 % (47/170) | n/a | n/a | n/a | 0.11 | n/a | n/a |
| LAD (mm) | 46.1 ± 8.9 | 45.5 ± 9.0 | -0.5 ± 7.1 | 0.27 | 42.9 ± 7.1 | 38.8 ± 7.9 | -4.1 ± 6.5 | <0.0001 | <0.0001 | <0.0001 | <0.0001 |
| LAD reduction ≥5%* ^a^ | n/a | 39 %  (88/227) | n/a | n/a | n/a | 64 %  108/170 | n/a | n/a | n/a | <0.0001 | n/a |
| Moderate/Severe MR ^a^ | 37 % (84/226) | 38 % (87/226) | 1.3 % | 0.67 | 39 %  66/168 | 20 % (33/168) | -18 % | <0.0001 | 0.67 | <0.0001 | <0.0001 |
| Moderate/Severe TR | 28 % (63/225) | 26 % (58/225) | -2.2 % | 0.47 | 22 % (37/168) | 17 % (28/168) | -5.4 % | 0.11 | 0.18 | 0.03 | 0.42 |
| TRPG (mmHg) | 33.2 ± 12.1 | 29.8 ± 13.8 | -2.6 ± 13.9 | 0.02 | 32.1 ± 14.3 | 23.8 ± 12.2 | -7.6 ± 14.6 | <0.0001 | 0.48 | <0.0001 | 0.005 |
| IVC (mm) | 16.5 ± 4.8 | 15.7 ± 4.7 | -0.8 ± 5.1 | 0.02 | 16.5 ± 5.3 | 14.1 ± 4.5 | -2.5 ± 5.7 | <0.0001 | 1.0 | <0.0001 | 0.004 |

^*^ LAD reduction was calculated as (LAD during index hospitalization-LAD at follow-up echocardiography)/ LAD during index hospitalization × 100 (%).

^#^ Delta was calculated for continuous variables according to the following equation: (the value at 6-month follow-up echocardiography) – (the value at baseline) and for binary variables according to the following equation: (the prevalence at 6-month follow-up echocardiography)– (the prevalence at baseline).

^a^ Risk-adjusting variables selected for the Cox proportional hazards regression model.

HFrecEF, heart failure with recovered ejection fraction; LVEDD, left ventricular end-diastolic dimension; LVESD, left ventricular end-systolic dimension; LVMI, left ventricular mass index; LVEF, left ventricular ejection fraction; LAD, left atrial diameter; MR, mitral regurgitation; TR, tricuspid regurgitation; TRPG, tricuspid regurgitant pressure gradient; IVC, inferior vena cava.

**Supplementary Table 2. Changes in echocardiographic parameters between HFrEF and HFmrEF**

|  | **HFrEF (N=270)** | | | | **HFmrEF (N=127)** | | | | **Between-groups comparison** | | |
| --- | --- | --- | --- | --- | --- | --- | --- | --- | --- | --- | --- |
| Variable | Baseline | Follow-up | Delta ^#^ | P value  (paired) | Baseline | Follow-up | Delta ^#^ | P value  (paired) | P value  (baseline) | P value  (follow-up) | P value  (delta) |
| LVEDD (mm) | 59.2 ± 8.8 | 55.5 ± 10.1 | -3.7 ± 6.2 | <0.0001 | 52.5 ± 6.7 | 50.1 ± 7.0 | -2.3 ± 5.5 | <0.0001 | <0.0001 | <0.0001 | 0.03 |
| LVESD (mm) | 50.8 ± 9.8 | 44.5 ± 12.0 | -6.4 ± 8.6 | <0.0001 | 40.5 ± 6.0 | 36.7 ± 8.2 | -3.9 ± 6.4 | <0.0001 | <0.0001 | <0.0001 | 0.001 |
| LVMI (g/m^2^) | 140.6 ± 34.5 | 125.2 ± 39.4 | -16.1 ± 29.4 | <0.0001 | 132.9 ± 38.0 | 115.7 ± 35.6 | -19.4 ± 30.8 | <0.0001 | 0.03 | 0.04 | 0.37 |
| LVEF (%) | 28.4 ± 7.3 | 39.4 ± 14.2 | 11.0 ± 13.3 | <0.0001 | 44.2 ± 2.9 | 50.4 ± 11.7 | 6.2 ± 11.3 | <0.0001 | <0.0001 | <0.0001 | 0.0002 |
| LVEF improvement ≥10%^$^ | n/a | 46 % (123/270) | n/a | n/a | n/a | 37 % (47/127) | n/a | n/a | n/a | 0.11 | n/a |
| LAD (mm) | 44.6 ± 8.5 | 42.0 ± 9.4 | -2.6 ± 7.3 | <0.0001 | 45.1 ± 7.8 | 44.1 ± 8.6 | -1.0 ± 6.5 | 0.06 | 0.53 | 0.03 | 0.03 |
| LAD reduction ≥5%* ^a^ | n/a | 54 % (146/270) | n/a | n/a | n/a | 39 % (50/127) | n/a | n/a | n/a | 0.006 | n/a |
| Moderate/Severe MR ^a^ | 39 %  (105/268) | 29 % (79/268) | -9.7 % | 0.001 | 36 % (45/126) | 33 % (41/126) | -3.2 % | 0.42 | 0.51 | 0.54 | 0.32 |
| Moderate/Severe TR | 25 %  (67/266) | 22 % (58/266) | -3.4 % | 0.23 | 26 % (33/127) | 22 % (28/127) | -3.9 % | 0.30 | 0.87 | 0.96 | 0.83 |
| TRPG (mmHg) | 33.2 ± 12.7 | 26.6 ± 13.8 | -6.1 ± 14.3 | <0.0001 | 31.8 ± 13.8 | 28.9 ± 12.7 | -1.8 ± 14.1 | 0.31 | 0.38 | 0.13 | 0.02 |
| IVC (mm) | 16.6 ± 5.1 | 14.6 ± 4.4 | -2.0 ± 5.4 | <0.0001 | 16.4 ± 4.9 | 15.8 ± 5.3 | -0.6 ± 5.4 | 0.09 | 0.73 | 0.03 | 0.02 |

^$^ LVEF improvement was an absolute difference in LVEF between during index hospitalization and at follow-up echocardiography.

^*^ LAD reduction was calculated as (LAD during index hospitalization-LAD at follow-up echocardiography)/ LAD during index hospitalization × 100 (%).

^#^ Delta was calculated for continuous variables according to the following equation: (the value at 6-month follow-up echocardiography) – (the value at baseline) and for binary variables according to the following equation: (the prevalence at 6-month follow-up echocardiography)– (the prevalence at baseline).

^a^ Risk-adjusting variables selected for the Cox proportional hazards regression model.

HFrEF, heart failure with reduced ejection fraction; HFmrEF, heart failure with mildly-reduced ejection fraction LVEDD, left ventricular end-diastolic dimension; LVESD, left ventricular end-systolic dimension; LVMI, left ventricular mass index; LVEF, left ventricular ejection fraction; LAD, left atrial diameter; MR, mitral regurgitation; TR, tricuspid regurgitation; TRPG, tricuspid regurgitant pressure gradient; IVC, inferior vena cava.

**Supplementary Table 3. Comparison of heart rate, left ventricular ejection fraction and left atrial diameter between atrial arrythmias and non-atrial arrythmias**

|  | **Non-atrial arrythmias (N=203)** | | | | **Atrial arrythmias (N=194)** | | | | **Between-groups comparison** | | |
| --- | --- | --- | --- | --- | --- | --- | --- | --- | --- | --- | --- |
| Variable | Baseline | Follow-up | Delta ^#^ | P value  (paired) | Baseline | Follow-up | Delta ^#^ | P value  (paired) | P value  (baseline) | P value  (follow-up) | P value  (delta) |
| Heart rate (bpm) | 70.7 ± 11.7 | 74.8 ± 12.9 | 4.5 ± 13.1 | <0.0001 | 72.3 ± 13.9 | 77.4 ± 16.3 | 4.8 ± 18.2 | 0.001 | 0.22 | 0.11 | 0.85 |
| LVEF (%) | 32.3 ± 9.4 | 42.8 ± 14.5 | 10.5 ± 13.3 | <0.0001 | 34.6 ± 9.8 | 43.1 ± 14.3 | 8.4 ± 12.3 | <0.0001 | 0.02 | 0.84 | 0.11 |
| LAD (mm) | 42.3 ± 7.2 | 39.1 ± 8.0 | -3.2 ± 6.7 | <0.0001 | 47.3 ± 8.6 | 46.4 ± 8.9 | -0.8 ± 7.3 | 0.12 | <0.0001 | <0.0001 | 0.0006 |

^#^ Delta was calculated for continuous variables according to the following equation: (the value at 6-month follow-up echocardiography) – (the value at baseline).

Atrial arrythmias included atrial fibrillation or atrial flutter.

LVEF, left ventricular ejection fraction; LAD, left atrial diameter.

**Supplementary Figure**

**Supplementary Fig. 1**

**Supplementary Fig. 2**

**Supplementary Fig. 3**

**Supplementary Fig. 4**

**Supplementary Fig. 5**

**Supplementary Fig. 6**

**Supplementary Fig. 7**

**Supplementary Fig. 8**

**Supplementary Fig. 9**

**Supplementary Figure legends**

**Supplementary Fig. 1. Study flowchart.**

Heart failure was classified according to LVEF at index hospitalization (HFpEF: LVEF ≥50%, HFmrEF: LVEF 40%–49% and HFrEF: LVEF <40%).

AHF, acute heart failure; KCHF, Kyoto Congestive Heart Failure; s-Cr, serum creatinine; LAD, left atrial diameter; LVEF, left ventricular ejection fraction; HFpEF, heart failure with preserved ejection fraction; HFmrEF, heart failure with mid-range ejection fraction; HFrEF, heart failure with reduced ejection fraction; HFrecEF, heart failure with recovered ejection fraction; LA, left atrium.

**Supplementary Fig. 2. Scheme of the present analysis.**

LVEF, left ventricular ejection fraction; HFrecEF; heart failure with recovered ejection fraction.

**Supplementary Fig. 3. Scattered plot for change in LVEF and change in LAD.**

LVEF, left ventricular ejection fraction; LAD, left atrial diameter.

**Supplementary Fig. 4. Detail of the primary outcome measure.**

HF, heart failure; CVD, cardiovascular death.

**Supplementary Fig. 5. Kaplan Meier curves for the primary outcome measure: HFimpEF versus non-HFimpEF.**

HFimpEF, heart failure with improved ejection fraction; HR, hazard ratio; CI, confidence interval.

**Supplementary Fig. 6. Kaplan Meier curves for the primary outcome measure by the combination of HFimpEF and left atrial reverse remodeling.**

HFimpEF, heart failure with improved ejection fraction; LA, left atrium; CI, confidence interval.

**Supplementary Fig. 7. Kaplan Meier curves for the primary outcome measure by three groups of change in left ventricular ejection fraction.**

LVEF, left ventricular ejection fraction.

**Supplementary Fig. 8. Clinical outcomes by three groups of change in left ventricular ejection fraction.**

LVEF, left ventricular ejection fraction; HR, hazard ratio; CI, confidence interval.

**Supplementary Fig. 9. Analysis for a composite of all-cause death or hospitalization for heart failure by the combination of three groups of change in left ventricular ejection fraction and left atrial reverse remodeling.**

LVEF, left ventricular ejection fraction; LARR, left atrial reverse remodeling; CI, confidence interval.
